# The multi-functional roles of forkhead box protein O in skin aging and diseases

**DOI:** 10.1016/j.redox.2021.102101

**Published:** 2021-08-13

**Authors:** Kyoung Mi Moon, Min-Kyeong Lee, Taehyeok Hwang, Chun Whan Choi, Min Soo Kim, Hyeung-Rak Kim, Bonggi Lee

**Affiliations:** a Department of Food Science and Nutrition, Pukyong National University, Nam-Gu, Busan, Republic of Korea; b College of Pharmacy and Research Institute of Pharmaceutical Sciences, Gyeongsang National University, Jinju, Republic of Korea; c Natural Product Research Team, Biocenter, Gyeonggido Business and Science Accelerator, Gyeonggi-Do, Republic of Korea; d Brain Science Institute, Korea Institute of Science and Technology (KIST), Seoul, 02792, Republic of Korea; e Division of Bio-Medical Science & Technology, KIST School, University of Science and Technology, Seoul, 02792, Republic of Korea

**Keywords:** FoxOs, Melanogenesis, Wound healing, Acne, Melanoma

## Abstract

Forkhead box, class O (FoxO) family members are multifunctional transcription factors that are involved in several metabolic processes, including energy metabolism, apoptosis, DNA repair, and oxidative stress. However, their roles in skin health have not been well-documented. Recent studies have indicated that FoxOs are important factors to control skin homeostasis and health. The activation or deactivation of some FoxO family members is closely related to melanogenesis, wound healing, acne, and melanoma. In this review, we have discussed the recent findings that demonstrate the relationship between FoxOs and skin health as well as the underlying mechanisms associated with their functions.

## 1 Introduction

Forkhead box, class O (FoxO) family members are multifunctional transcription factors that integrate various signaling pathways in transcriptional networks, ensuring the control of cell and tissue homeostasis over time and in response to environmental challenges [1]. Evolutionarily, conserved FoxOs in mammals are composed of four proteins, including FoxO1, FoxO3a, FoxO4, and FoxO6. Diverse cell and animal research studies have revealed that the signaling molecules transcriptionally regulated by FoxOs include, but are not limited to, oxidative stress, apoptosis, cell cycle, energy metabolism, and DNA repair [[2], [3], [4], [5]]. FoxOs are controlled by nutritional and physiological factors, including cytokines, insulin, insulin-like growth factors (IGFs), oxidative stress, and macro- and micro-nutrients [6,7].

Oxidative stress plays essential roles in both intrinsic and extrinsic aging processes; therefore, numerous efforts have been made to relieve oxidative stress in multiple organs and cells. FoxOs have been reported to ameliorate the oxidative stress produced by multiple stimuli, including oxidants, inflammatory cytokines, and ultraviolet (UV) light [8,9]. When the function of FoxO4 in regulating the cellular redox reactions was studied in A14, C2C12, or 293T cells, oxidative stress induced through hydrogen peroxide (H_2_O_2_) treatment was found to activate the small GTPase Ral followed by the c-Jun NH2-terminal kinase (JNK)-dependent activation of FoxO4 [9]. The activation of FoxO4 protects the cell against oxidative stress by upregulating manganese superoxide dismutase (MnSOD) and catalase [9]. Small interfering RNA (siRNA)-mediated decrease in the expression levels of FoxO1 and FoxO3 in chondrocytes significantly elevates the susceptibility to cell death stimulated by the oxidant, tert-butyl-hydroperoxide, which is associated with the decreased levels of antioxidant proteins and autophagy-associated proteins [8]. FoxO6 has been reported to regulate oxidative stress in skin cells. Adenovirus-mediated FoxO6 knockdown and activation elevates and reduces the oxidative stress, respectively, by controlling the antioxidant gene expression in B16F10 cells [10]. Therefore, FoxOs are important transcription factors that control the cellular redox homeostasis.

The skin is the most outer part of our body that acts as a protective barrier against environmental pollution and UV irradiation. The skin consists of mainly two parts the epidermis and dermis, having a variety of cell types. Keratinocytes are the main cell type in the epidermis. The primary function of keratinocytes, especially apoptotic keratinocytes, is the formation of the skin barrier in the stratum corneum against environmental challenges, such as UV irradiation, heat, and water loss as well as the presence of microbes, including fungi, parasites, bacteria, and viruses [1,11]. Melanocytes located in the epidermis can produce melanin and transfer it to keratinocytes through the dendrites, stimulating skin darkening. Although melanogenesis is a defense mechanism against various environmental challenges, the overproduction of melanin causes undesired pigmentation [12,13]. Furthermore, many variants of genes associated with melanogenesis and skin darkening are epidemiologically related with an increased risk of skin cancers [14]. The epidermis also includes Merkel cells that play an essential role in tactile sensation and Langerhans cells that mediate the antigen-presenting functions. The epidermis and dermis are separated by a thin sheet of proteins, called the basement membrane, which are secreted from both keratinocytes and fibroblasts, and form a primary cell type in the dermis [1]. Fibroblasts produce several proteins and glycans of the extracellular matrix surrounding them and other dermal cells, including adipocytes and macrophages. Both the epidermis and dermis cooperate in the development of skin adjuncts, including hair follicles and sebaceous glands [1,11].

As the skin is inevitably exposed to a variety of harmful factors derived from the environment and chemicals, the maintenance of skin homeostasis is not easy to achieve. Especially, UV continuously attacks the skin, potentially leading to inflammation, dermal connective tissue damage, photoaging, and skin cancers [15]. Among several factors, oxidative stress, in which the elevation of oxidants exceeds the antioxidant defense system capacity, results in collagen destruction, disorganization of collagen fibers, and impairment of skin cell functions, contributing to various skin disorders, including cancers [16].

Although various studies related to FoxOs have been performed, the roles of FoxOs in skin health have not yet been extensively investigated. Nevertheless, recent *in vitro* and *in vivo* studies have shown that FoxOs play diverse roles in various processes, including melanogenesis, anti-oxidative effects, wound healing, acne, and melanoma, to maintain the skin health. In this review, we have discussed the association between the FoxO subfamily members, including FoxO1, FoxO3a, FoxO4, and FoxO6, and skin health.

## 2 Roles of FoxOs in melanogenesis

Although the mRNA expression levels of various FoxOs were examined in melanogenic cell models, only FoxO3 and FoxO6 were deeply studied for their anti-melanogenic effects [10,17]. When the association between melanogenesis and the mRNA expression levels of FoxO1, FoxO3a, and FoxO4 were tested using lightly pigmented, moderately pigmented, and darkly pigmented primary melanocytes, only FoxO3a expression was found to be elevated more than 10 fold than the other FoxOs, and its mRNA expression level reduced with increasing degree of pigmentation, indicating that FoxO3a is inversely associated with melanin levels [17].

### 2.1 FoxO3a and melanogenesis

The phosphoinositide 3-kinase/serine-threonine kinase (PI3K/AKT) signaling pathway is a primary regulator of the FoxO family members [18]. FoxO3a activation by PI3K inhibitors, LY294002 and wortmannin, significantly decreases the levels of melanin and pigment-related proteins, including tyrosinase, tyrosinase-related protein 1, and dopachrome tautomerase, in primary-cultured melanocytes [17]. On the other hand, the siRNA-mediated knockdown of FoxO3a in human melanoma MNT1 cells highly elevates the pigmentation and mRNA expression levels of melanogenesis-related genes, suggesting that FoxO3a activation prevents melanogenesis. To further study the effects of PI3K inhibitors on FoxO3a regulation, the nuclear localization of FoxO3a was examined in MNT1 cells. PI3K inhibitors stimulate the translocation of FoxO3a in the nucleus and the protein levels of melanogenic genes, including the microphthalmia-associated transcription factor, tyrosinase, tyrosinase-related protein, and dopachrome tautomerase 1 [17]. However, the FoxO3a nuclear translocation is not observed when FoxO3a harbors a deletion in the nuclear localization signaling process. These data suggest that the nuclear localization of FoxO3a is necessary for its anti-melanogenic effect [17].

**Fig. 1:**
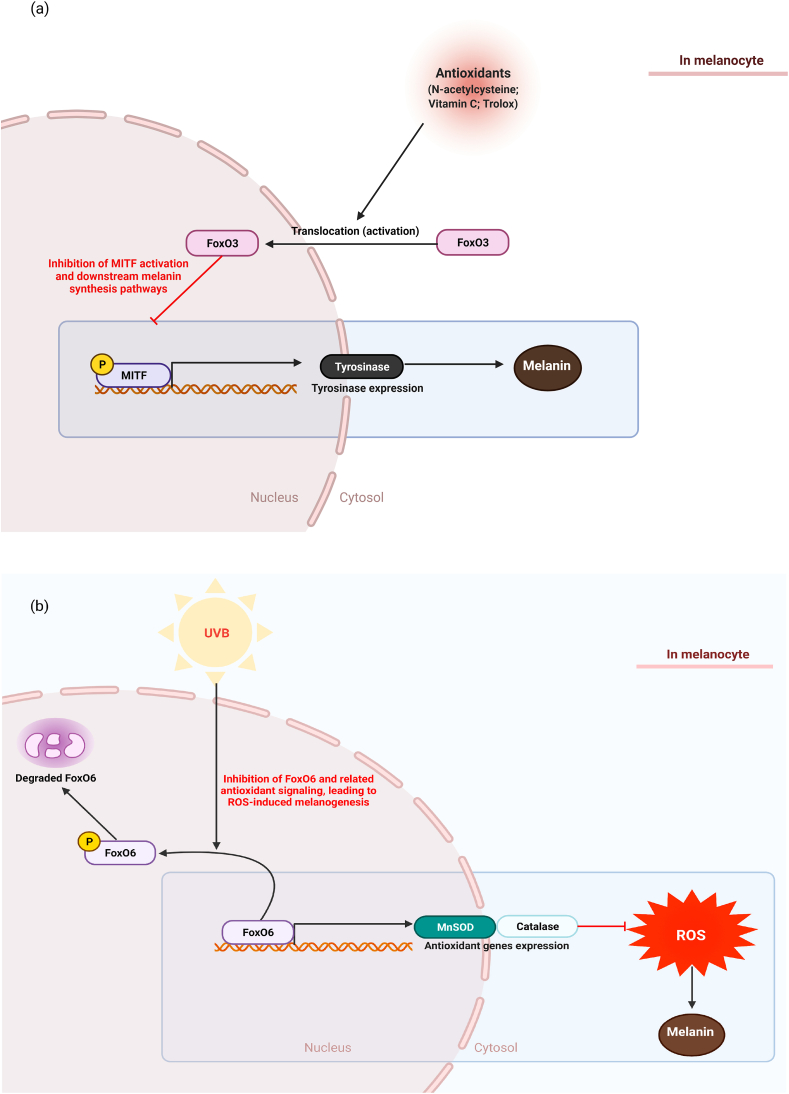
The roles of FoxOs in skin pigmentation. (a) FoxO3a down-regulates a melanogenic transcription factor MITF followed by a decrease in tyrosinase expression, leading to the suppression of melanin production. FoxO3 activation is necessary for the anti-melanogenic effects of antioxidants including N-acetylcysteine, vitamin C, and Trolox although underlying mechanisms need to be further elucidated. (b) FoxO6 decreases in the skin of UVB-exposed and biological aging mice that show increased oxidative stress and skin pigmentation. FoxO6 inhibits UVB-induced melanogenesis through the elevation of the intracellular antioxidant defense system via up-regulating MnSOD and catalase. As a result, oxidative stress is relieved and melanin synthesis pathways are inhibited.

The role of FoxO3 in antioxidant-mediated depigmentation effects was investigated using MNT1 cells with mature stage III and IV melanosomes that produce melanin in response to various stimuli [17]. When MNT1 cells were treated with several antioxidants, including trolox, vitamin C, and N-acetylcysteine, melanin accumulation was reduced with FoxO3 nuclear translocation. However, this phenomenon disappeared when FoxO3a was deactivated by siRNA-mediated knockdown, suggesting that the mechanisms underlying the anti-melanogenic effects of antioxidants are not only due to the direct scavenging of reactive oxygen species, but also due to the activation of FoxO3a. Although further studies are necessary to confirm this, FoxO3 may potentially orchestrate the antioxidant-mediated depigmenting effects (Fig. 1a).

### 2.2 FoxO6 and melanogenesis

Another research study tested the roles of FoxO6 in skin pigmentation [10]. FoxO6 shows fundamental differences from other FoxO family members as it exhibits the lowest protein homology (~30%) in amino acid sequence compared to those of the other FoxOs [7,19]. More specifically, the similarity of FoxO6 to the other FoxOs is highest in the forkhead domain, while the other regions are moderately to highly conserved. The homology is striking in two regions associated with phosphorylation by protein kinase B (PKB) via the well-known PI3-kinase/PKB pathway. Remarkably, a third conserved region, including a stretch of four phosphorylation sites as observed in the other FoxOs, such as Daf16, does not exist in FoxO6 [7,19].

Based on transfection studies using FoxO6 coupled to GFP, high nuclear localization even after stimulation with growth factors was observed unlike the predominant cytosolic localization of other family members, possibly due to the lack of the consensus motif that stimulates the nuclear export signal [19]. Nevertheless, insulin signaling can suppress FoxO6 activity by directly stimulating FoxO6 phosphorylation [18].

Another study showed that FoxO6 plays an important role in suppressing intrinsic- and UV-induced melanogenesis. In this study, UV exposed and intrinsically aged mice were used to investigate the relationship between FoxO6 and skin aging. The researcher revealed that the activation of FoxO6 was notably reduced in the skin of intrinsically aged mice and UVB-irradiated HRM-2 hairless mice that showed the elevation of cutaneous pigmentation [10]. Also, the decrease in FoxO6 activity was related to the increase in oxidative stress in the skin of these mouse amodels. To further study the effect of FoxO6 on oxidative stress and pigmentation, a melanogenic B16F10 cell model was used with adenovirus-mediated FoxO6 manipulation. FoxO6 knockdown by adenoviral delivery of siRNA significantly elevated the pigmentation and melanogenesis-related signaling pathways in the cells, without any stimulation [10]. Meanwhile, adenoviral FoxO6 activation highly decreased the pigmentation in UVB-irradiated B16F10 cells. These phenomena are closely related to the upregulation of antioxidant genes, such as *MnSOD* and catalase. Therefore, UVB-induced oxidative stress is ameliorated by FoxO6 activation. Besides, the treatment with ascorbic acid reversed the increased pigmentation derived from the FoxO6 knockdown, suggesting that the reduced antioxidant capacity may lead to pigmentation in the FoxO6 knockdown condition [10]. In the same study, the authors also investigated the upstream signaling of FoxO6 related to pigmentation. It appears that FoxO6 phosphorylation by AKT is important for the FoxO6-mediated regulation of pigmentation, evidenced by the decrease in FoxO6 activity and increase in melanogenesis by PI3K/AKT inhibitor treatment. These data suggest that FoxO6 is a transcription factor upregulating antioxidant gene that suppresses the oxidative stress-induced melanogenesis (Fig. 1b) [10].

Several studies related to FoxO3a and FoxO6 and pigmentation raised pertinent questions about the mechanisms underlying FoxO-induced anti-melanogenic effects. Although more detailed molecular studies are necessary to reveal the downstream signaling pathways regulated by FoxOs for their anti-melanogenic effects, the mechanism underlying the FoxO6-mediated anti-melanogenic effect appears to be slightly different with FoxO3 [10,17] because the increased pigmentation with FoxO6 knockdown was reversed by ascorbic acid treatment [10], indicating that FoxO6 may not be an essential factor for ascorbic acid-mediated anti-melanogenic effect [10], while FoxO3 appears to be necessary for this effect [17]. When the two different studies mentioned above were analyzed, it was determined that FoxO6 exerts anti-melanogenic effects partly through the elevation of the cellular antioxidant defense system, thereby inhibiting the UVB-induced oxidative stress. On the other hand, FoxO3a may interact with other melanogenic proteins to regulated pigmentation and is likely an important factor for antioxidants to prevent melanogenesis (Fig. 1a-b) [17].

## 3 Roles of FoxOs in melanoma

Melanoma is a skin malignancy induced by genetic mutations in melanocytes, the pigment-producing cells, which can be found in the skin, eye, leptomeninges, and inner ear [20,21]. Although the morbidity rate of melanoma is about 1 % of skin cancers, which is less common than other skin cancers, it is the most aggressive and deadliest form, accounting for nearly 73 % of skin cancer-related deaths [22,23]. Although surgical resection is an effective treatment for localized disease, the average survival of patients with metastatic malignant melanoma, post-treatment with radiation and chemotherapy drugs, is only 6–9 months [24]. Therefore, it is necessary to understand the underlying pathological mechanisms and new molecular targets to develop effective therapeutic strategies.

**Fig. 2:**
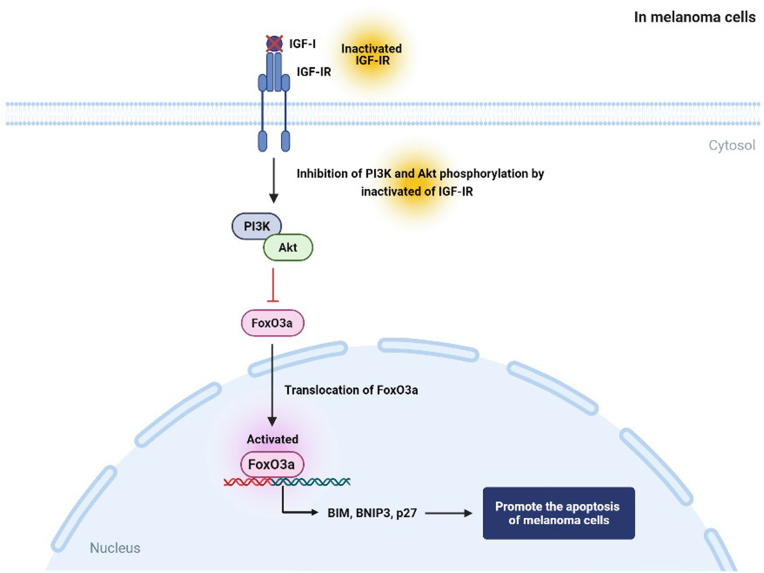
The roles of FoxO3a in melanoma. Nuclear translocation of FoxO3a induced by inactivation of the IGF-I/PI3K/Akt signaling pathway promotes the expression of apoptotic proteins such as BIM, BNIP3, and p27, leading to apoptosis of melanoma cells.

In melanoma, strong resistance to apoptosis stimulation and high metastasis rates are closely related to the activity of FoxO3a [25,26]. FoxO3a is also known as an essential tumor suppressor and has been shown to regulate gene expression associated with cell survival, apoptosis, migration, and invasion in melanoma [[25], [26], [27], [28], [29]]. The function of FoxO3a is negatively regulated by IGF-I/PI3K/Akt signaling, and phosphorylation and subsequent nuclear exclusion of FoxO3a by these kinases promote the survival of tumor cells by inhibiting the biosynthesis of proapoptotic FoxO3a target genes [30]. Nuclear FoxO3a induces apoptosis via the upregulation of apoptotic proteins, such as BIM, B-cell lymphoma (BCL)-2-interacting protein 3 (BNIP3), and p27, and inhibition of anti-apoptotic molecules, such as the Fas-associated death domain-like interleukin-1β-converting enzyme (FLICE)-like inhibitory protein (FLIP) and BCL-XL (Fig. 2) [[31], [32], [33]]. A study showed that IGF-I promotes phosphorylation and nuclear exclusion of FoxO3a through activation of the PI3K/Akt signaling pathway in melanoma cells [25]. In contrast, overexpression of the non-phosphorylable mutation of FoxO3a (FoxO3a-TM), which is constitutively localized to the nucleus, reduced the mRNA expression of survivin, a member of the inhibitor of apoptosis protein, and promoted apoptosis of melanoma cells [25]. Also, NOVA1 (RNA-binding proteins) and tribbles pseudokinase 2 (TRIB2) (mammalian homolog of the Drosophila gene tribbles) have been shown to contributes to the maintenance of the malignant phenotype of melanoma cells through inhibition of FoxO3a activity [26,34]. In vitro studies demonstrated that NOVA1 and TRIB2 transcript levels were up-regulated in melanoma malignancies and melanoma cell lines [26,34]. In particular, knockdown of NOVA1 and TRIB2 has been shown to inhibit melanoma cell proliferation, migration, and invasion partly through promoting nuclear translocation of FoxO3a [26,34].

A study confirmed that 5-aza-2′-deoxycytidine (5-aza), which has attracted attention as a promising therapeutic agent for solid cancer, exerts anti-tumor effects in melanoma [35]. In human and canine melanoma cells, 5-aza promotes apoptosis by upregulating the mRNA expression of the tumor necrosis factor-alpha (TNF-α) via the activation of FoxO1 resulting from the inactivation of Akt [35]. FoxO3a was recently found to regulate aerobic glycolysis by modulating the expression of sirtuin 6 (SIRT6), which is recognized as a key regulator of cellular metabolism in melanoma [36]. It is suggested that FOXO3a expression is positively correlated with SIRT6 expression and that the FOXO3a-SIRT6 regulatory axis inhibits glucose metabolism and tumor cell proliferation in melanoma [36].

Melanoma is also regulated by microRNAs (miRNAs)-mediated FoxO regulation. Studies showed that FoxO1 and FoxO3a are a target of regulation by melanoma-related miRNAs [[37], [38], [39], [40]]. For example, a study indicated that miRNA-135a promotes cell proliferation, tumorigenicity, and cell cycle progression by transcriptionally downregulating FoxO1 in malignant melanoma cells [38]. miRNA-155 induces cell survival in melanoma, and its anti-apoptotic function is mediated by inhibition of nuclear translocation of FoxO3a [40]. Also, overexpression of miR-182 promotes metastasis and survival of melanoma cells *in vitro* by transcriptionally suppressing FoxO3a and the melanocyte inducing transcription factor (MITF). In contrast, the enhanced expression of FoxO3a blocks the pro-invasive effect of miR-182 by activating BIM, a pro-apoptosis factor [37]. Furthermore, overexpression of miRNA-194 activates FoxO3a via the inhibition of the PI3K/Akt signaling pathway, thereby inhibiting proliferation and inducing apoptosis of melanoma cells [39]. These observations indicate that activating FoxO1 and FoxO3a may be beneficial in the treatment of melanoma (Fig. 2) although further studies are necessary to reveal detailed mechanisms. The close relationship between FoxOs and drug resistance are also reported [41,42]. Although several studies have revealed that FoxO1 and FoxO3a play important roles in increasing the drug resistance in various diseases, such as ovarian cancer and chronic myelogenous leukemia [41,42], drug resistance studies involving FoxO1 and FoxO3a in melanoma have not yet been conducted, so further studies are needed to investigate this.

## 4 Roles of FoxOs in skin regeneration

Acute skin damage quickly induces tissue repair mechanisms with an inflammatory response for host defense. Generally, the skin wound healing process includes four steps: coagulation, inflammation, proliferation/migration, and remodeling [43]. Bleeding is stopped by the contraction of blood vessels and the coagulation of platelets. In the inflammation step, a lot of immune cells, including neutrophils and macrophages, are transferred to the damaged tissues to protect against microbes and contribute to the formation of granulation tissue, followed by endothelial cells to form new blood vessels [[44], [45], [46]]. Angiogenesis is triggered by multiple signals and induces the endothelial cell proliferation. Epithelial cells repair the connective tissue and move over the newly forming granulation tissue to cover the wound site [47]. Finally, in the re-epithelialization step, the wound area of the skin is partially remodeled by the removal of excess extracellular matrix (ECM) at the scar area [48,49]. Skin scarring caused by an abnormal healing process is associated with aging, diabetes, malnutrition, chemotherapy, and hereditary diseases [[50], [51], [52], [53], [54]].

### 4.1 Negative roles of FoxO1 in wound healing

**Fig. 3:**
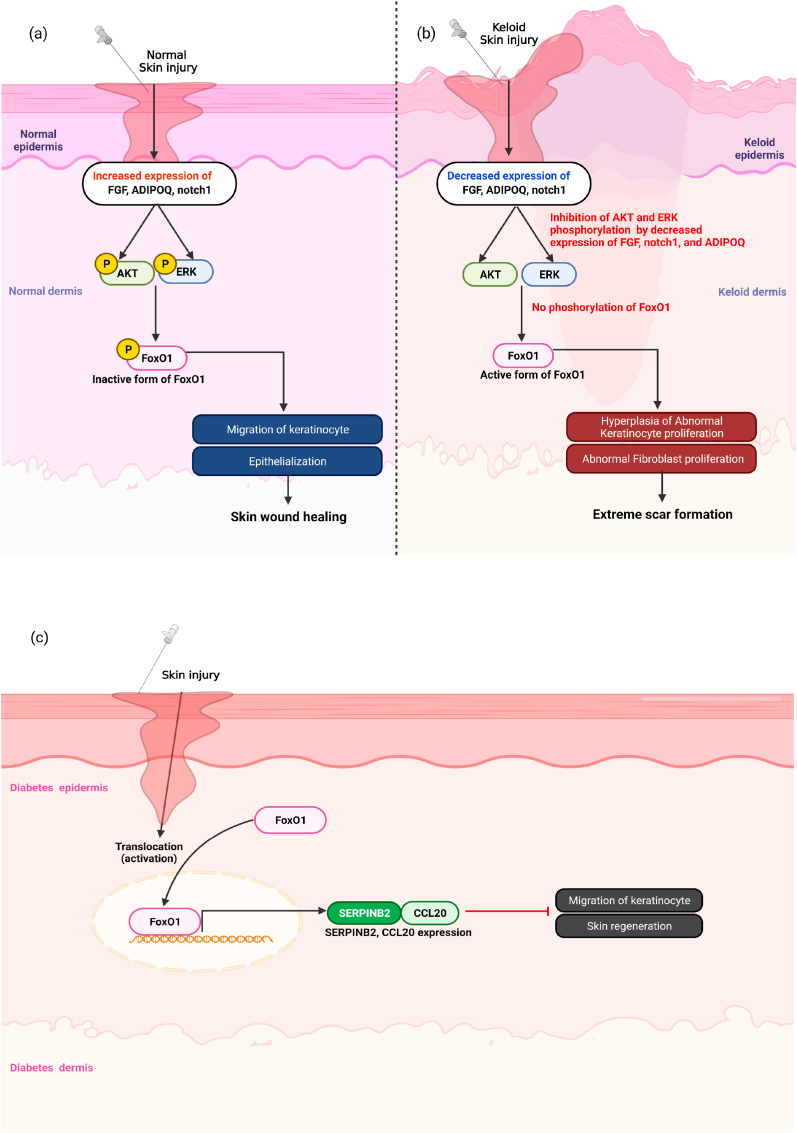
Negative roles of FoxO1 in wound healing. (a) When normal skin is injured, increased expression of FGF2, ADIPOQ, and notch1 induces inactivation of FoxO1 by phosphorylation of ERK/AKT signaling pathway, leading to the epithelialization and migration of keratinocyte at wound healing. (b) In the case of keloid skin, reduced expression of FGF2, ADIPOQ, and notch1 decreased phosphorylation of AKT and ERK followed by increase activation of FoxO1. An increase of FoxO1 produces hyperplasia of abnormal keratinocyte proliferation and abnormal collagen. therefore, FoxO1 induces an extreme scar formation. (c) In diabetic wound healing, FoxO1 nuclear localization inhibits keratinocyte migration by upregulating SERPINB2 and CCL20 transcription.

FoxO regulates the homeostasis in the immune system and the inflammatory response [55,56]. The deficiency of FoxO1 (FoxO1−/−) in embryonic mice is lethal due to abnormal vascular generation [57,58]. To verify whether FoxO1 affects skin wound healing, heterozygous FoxO1-deficient (FoxO1+/−) mice were generated by partial knockout of FoxO1 [59]. FoxO1 ± mice showed an acceleration of skin wound healing through a decrease in the inflammatory response and increased keratinocyte migration and collagen degradation [59]. As molecular factors associated with these phenotypes, the expression levels of the fibroblast growth factor 2 (*FGF2*), adiponectin (*ADIPOQ*), and *Notch1* genes associated with cell migration and proliferation signals were significantly upregulated at the wound area in the FoxO1 ± mice. FGF2 and ADIPOQ are important regulators of the extracellular signal‐regulated kinase (ERK)-1/2 and AKT as they inactivating the signaling of FoxO1, which stimulates cell migration and proliferation in wound healing (Fig. 3a) [[60], [61], [62]]. In FoxO1 ± mice, the phosphorylation of ERK 1/2 and AKT is extremely elevated. Therefore, it appears that the activation of ERK and AKT pathways in FoxO1 ± mice contributes to the epithelialization and migration of keratinocytes to facilitate wound healing [59]. FGF2 treatment enhances the ERK pathway in FoxO1 knocked down fibroblasts and local treatment of FGF2 reduces scarring at the incision site in humans [50,60,61,63]. In addition, the decrease in FoxO1 activity in the skin wound areas may contribute to the reduction of scarring by activating FGF2 signaling [59,63]. To better understand the pathophysiological significance of FoxO1 function in the skin, the most extreme scar phenotype in patients was studied in keloid scarring [59]. Keloids are most frequently found in individuals of Africa-American descent [64]. To verify the expression of FoxO1, samples of human keloid tissues were collected from African American and Japanese patients at the Nagasaki University Hospital, and it was found that the FoxO1 expression levels were increased more in the African Americans than the Japanese patients [59]. The expression of FoxO1 is highly increased in fibroblasts and inflammatory cells in keloid scars and the gene expression levels of several wound growth factors, including *FGF2*, *Notch1*, and *ADIPOQ*, are also decreased in keloid scars [59,63]. It is speculated that the expression of FoxO1 is increased due to the downregulation of several growth factors through the ERK/AKT pathway [65,66] (Fig. 3b).

FoxO1 is negatively associated with wound healing processes in diabetic mice. FoxO1 is upregulated at the wound sites in diabetic animals [[67], [68], [69], [70]]. To verify the function of FoxO1 in wound healing in diabetes, diabetic characteristics were induced in keratinocyte-specific FoxO1-deficient mice by multiple i.p. injections of streptozotocin (40 mg/kg). Keratinocyte-specific FoxO1-deficient mice without diabetes exhibited delayed wound healing [71], but the same mouse model with diabetes had the opposite effect [72]. In further *in vitro* experiments, wound area was remarkably increased in FoxO1-deficient keratinocyte incubated under normal glucose media, but the wound area was remarkably decreased in a high glucose media [72], suggesting that the roles of FoxO1 in wound healing are different depending on the concentration of glucose. As potential factors related to the different results between the low and high glucose media, FoxO1 promotes keratinocyte migration by upregulating TGF-1β when keratinocytes are wounded in normal glucose media, but in high glucose media, FoxO1 binds to the promoter regions of the serpin family E member 2 (SERPINE2) and C–C motif chemokine ligand 20 (CCL20), factors that inhibit skin wound healing, instead of the transforming growth factor- 1 beta (TGF-1β), leading to suppression of keratinocyte migration [72]. Therefore, nuclear localization of FoxO1 could have positive or negative effects on keratinocyte migration, depending on the downstream target genes in normoglycemic or diabetic conditions (Fig. 3c).

### 4.2 Positive roles of FoxO1 in wound healing

**Fig. 4:**
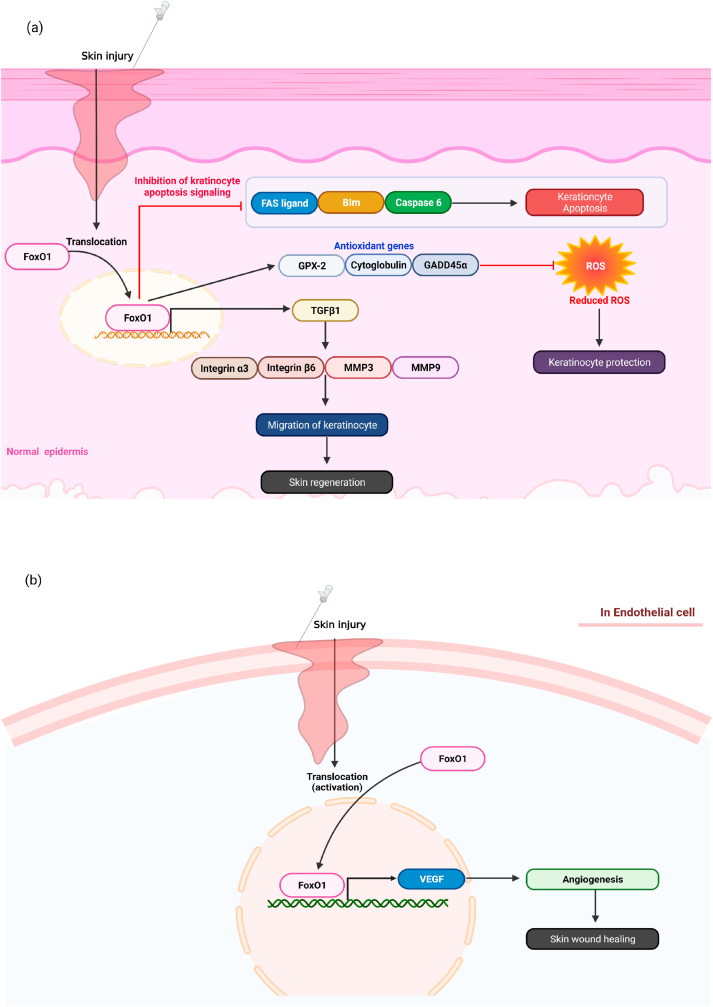
Positive roles of FoxO1 in wound healing. (a) In normal glucose levels, FoxO1 increases keratinocyte migration by upregulating TGF-1β transcription and its target genes including integrin-a3, integrin-B6, mmp3, and mmp9. In addition, FoxO1 protects cells by up-regulating the expression of antioxidant genes including GPX-2, Cytoglobulin, and GADD45α, and inhibiting the expression of apoptosis genes such as FAS ligand, Bim, Caspase 6 during wound healing processes. (b) Besides, FoxO1 up-regulates an expression of vascular endothelial growth factor VEGF in endothelial cells, leading to an increase of skin wound healing in endothelial cells.

FoxO1 in keratinocytes plays positive roles in wound healing processes. FoxO1 reportedly regulates cell migration in skin regeneration [59,73,74]. siRNA-mediated depletion of FoxO1 decreases wound healing in primary keratinocytes and the keratinocyte-specific FoxO1-deficient mice show remarkably delayed wound closure of the skin epidermis [71]. FoxO1 regulates a variety of gene involved in cell migration and remodeling, without affecting cell proliferation, as evidenced by the decrease in the mRNA levels of cell migration regulated-genes, including *TGF-β1*, integrin β6, integrin α3, matrix metalloproteinase (*MMP)-9*, and *MMP3*, and cell remodeling genes, such as collagen 4, in FoxO1-deficient keratinocytes [71]. Of these genes regulated by FoxO1, TGF-β1 may exhibit an important mechanism underlying the impaired keratinocyte migration by FoxO1 deficiency as TGF-β1 treatment reversed the keratinocyte migration in FoxO1-deficient keratinocytes. Consistently, TGF-β1 treatment upregulated the TGF-β1 downstream target genes related to cell migration, including integrin β6 and integrin α3 [71]. Further studies using promoter analysis, CHIP assay, and luciferase reporter assays indicate that FoxO1 directly interacts with TGF-β1 promoter in primary keratinocytes, suggesting that FoxO1 stimulates keratinocyte migration at least partially by inducing TGF-β1 transcription and upregulating the downstream target genes related cell migration (Fig. 4a).

FoxO1 also stimulates cell migration by relieving oxidative stress and apoptosis in keratinocytes [68,75,76]. To investigate the functions of FoxO1 in oxidative stress and apoptosis, the terminal deoxyribonucleotidyl transferase (TDT)-mediated dUTP nick-end labeling (TUNEL) and scratch-wound assays were performed. When FoxO1-deficient keratinocytes and keratinocyte-specific FoxO1-deficient mice were wounded, apoptosis was notably higher than a control group, and mRNA levels of apoptotic genes, such as caspase 6, FAS ligand, and Bim were increased [71]. Also, FoxO1 deficiency elevated ROS and decreased mRNA levels of the antioxidant genes, including glutathione peroxidase 2 (GPX-2) and cytoglobulin, and mRNA levels of DNA repair gene, such as growth arrest and DNA damage-inducible 45 alpha (*GADD45α*), leading to a decrease in the number of keratinocyte in wound area [71]. These findings indicate that FoxO1 is necessary to prevent keratinocyte apoptosis and oxidative stress (Fig. 4a).

Vascular endothelial growth factor (VEGF) has a positive effect on angiogenesis as well as granulation tissue formation, re-epithelialization, and collagen deposition in the skin wound healing process [77,78]. The keratinocyte-specific deletion of FoxO1 reduces the endothelial cell proliferation and angiogenesis by decreasing the expression of VEGFA in mucosal and skin injury. Besides, wound closure is extremely damaged in the keratinocyte-specific deletion of FoxO1. These data suggest that FoxO1 activation stimulates the production of VEGFA and re-epithelialization both *in vivo* and *in vitro* (Fig. 4b) [79]. 10.13039/100014337Furthermore, the reduced angiogenesis was reported by FoxO1 inhibitor treatment in damaged human skin, supporting the important roles of FoxO1 in human skin damage, which may be used in skin wound therapy [24]. FoxO1 interacts directly with the VEGFA promoter when analyzed by promoter analysis and the chromatin immunoprecipitation (CHIP) assay, and luciferase reporter assays showed that overexpression of FoxO1 increases VEGFA transcription. Thus, FoxO1 may be necessary for normal angiogenesis in skin wound healing (Fig. 4b) [79]. Considering both negative and positive effects of FoxO1 on wound sites, the roles of FoxO1 in wound healing may be different depending on the skin cell types and the blood glucose concentration.

### 4.3 FoxO3 and wound healing

Another study used an unbiased approach to find factors related to skin wound healing processes. The study analyzed 100 genes, the most differentially expressed between skin wounds and skin non-wound using overrepresented and co-occurring transcription factor-binding site (TFBS) [80]. FoxO1, FoxO3, and FoxO4 were found in the promoter regions of the 70 genes out of 100 between the wounded and non-wounded skin. In addition, FoxO3 was found in the promoter region of most genes involved in the wound healing process. Based on these data, the target genes of FoxO3 related to wound healing include innate immunity genes; antimicrobial peptides, such as human β -defensin 2 (DEFB4), Psoriasin (S100A7), calgranulin A and B (S100A8, S100A9), and koebnerisin (S100A7A); and pro-inflammatory cytokines, such as interleukin (IL)-20, IL-24, IL-6, and IL-8. Also, according to an analysis of the mRNA expression level of the FoxOs transcription factor, FoxO3 showed the highest expression level during the wound healing process in human and mouse skin [80]. When the roles of FoxO3 in wound healing were examined in human primary keratinocytes, the treatment of a constitutively active form of FoxO3, in which phosphorylation sites (Thr32, Ser253, and Ser315 on FoxO3) were substituted by alanine, decreased wound closure in human primary keratinocytes. Meanwhile, FoxO3 knockdown exhibited the opposite effects on keratinocytes and consistently, FoxO3 KO mice showed accelerated rate of skin wound healing [50]. Therefore, the inhibition of FoxO3 may be beneficial for wound healing processes.

### 4.4 Upstream signaling pathways of FoxOs for wound healing

The upstream signaling pathways of the FoxO family involved in the wound healing process are largely unknown. Nevertheless, some studies showed that the growth factor and cytokine signaling pathways were associated with the regulation of FoxOs. FoxO activity in type 2 diabetes is less sensitive to growth factors needed for wound healing. As the FoxO activity is negatively controlled by insulin receptors and AKT activation, it is difficult to regulate effective wound healing in diabetic patients [81]. Growth factors and epidermal growth factor receptor (EGFR) signals in the normal wound healing process are likely to cause cytosolic localization of FoxO (an inactive form of FoxO) via the downstream activation of AKT and the serum-and glucocorticoid-inducible protein kinase (SGK) [[82], [83], [84], [85], [86]]. TNF-α signaling pathways are related to decreased wound healing in type 2 diabetic mice (db/db mice). The increase in TNF-α, fibroblast apoptosis, FoxO1 activity, and caspase-3/7 activity was found in diabetic wounds. The suppression of TNF-α ameliorated the healing processes in diabetic mice and elevated the fibroblast density, which is likely explained by the reduction in fibroblast apoptosis and elevated proliferation with the blocking of TNF-α [70]. Interestingly, further *in vitro* studies exhibited that TNF-α upregulated the mRNA levels of genes related to apoptosis and inflammation, while FoxO1 knockdown reversed these effects [70]. It is likely that TNF-α delays wound healing processes, at least partly through the activation of FoxO1 that controls a variety of genes associated with apoptosis and inflammation [70].

## 5 Roles of FoxOs in acne

Acne, the most common inflammatory skin disease, is a chronic and multifactorial disorder of the sebaceous glands associated with hair follicles. Its development is related to exposome factors, such as heredity, occupation, climate, lifestyle (particularly nutrition), exposure to drugs and pollutants, and psychosocial issues [87]. Exposome factors affect the natural skin barrier and associated micro-organisms, resulting in enhanced sebaceous gland activity and hyperseborrhea, abnormal follicular differentiation, increased cornification, bacterial hypercolonization, and follicular and perifollicular inflammation [88]. These factors interact and cause chronic inflammation localized to the pilosebaceous unit [89]. Acne has prevalence rates of over 85 % in adolescents, causing anxiety, anger, depression, decreased self-esteem, facial scarring, and impaired social interactions. These degrade the quality of life of patients with acne [90,91].

The recent viewpoint on the pathogenesis of acne underlines that a western diet (WD) characterized by hyperglycemic carbohydrates and dairy protein consumption increases the insulin/IGF-1 signaling, thereby promoting acne [92]. Increased growth factors during puberty and sustained growth factor signals due to WD stimulate the PI3K/Akt cascade in the sebaceous glands of patients with acne, increasing the nuclear phosphorylation of FoxO1 and their export into the cytoplasm [[93], [94], [95]]. A study has demonstrated the potential association of IGF-I, mammalian target of rapamycin (mTOR), and FoxO1 in the pathogenesis of acne via immunohistochemical detection [93]. Nuclear deficiency and cytoplasmic expression of FoxO1 are increased in the sebaceous glands of patients with acne compared to the healthy control group, which is associated with increased serum IGF-I levels and activation of the mammalian target of rapamycin complex 1 (mTORC1) [93].

**Fig. 5:**
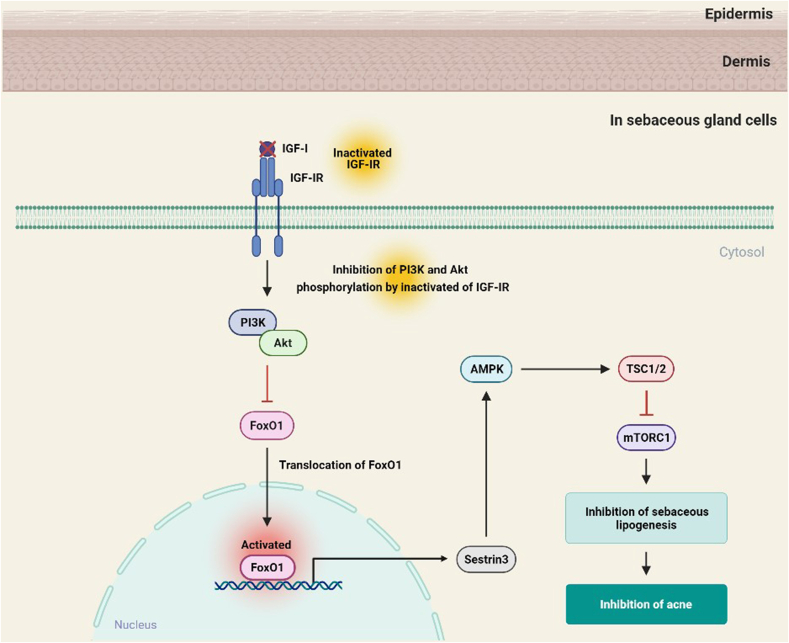
The roles of FoxO1 in acne. Nuclear translocation of FoxO1 induced by inactivation of the IGF-I/PI3K/Akt signaling pathway activates AMPK, a major negative regulator of mTORC1, through the upregulation of Sestrin-3. Activated AMPK suppresses acne by reducing the expression of mTORC1 in a TSC2-dependent manner.

FoxO1 is a pivotal inhibitor of mTORC1 and acts as a rheostat, regulating the activity of mTORC1 [96]. FoxO1 phosphorylated by the PI3K/Akt cascade induces the activation of mTORC1 by dissociating the tuberous sclerosis complex 1/2 (TSC1/TSC2) via the downregulation of sestrin3 and adenosine monophosphate-activated protein kinase (AMPK) [97]. Activated mTORC1 further increases the expression of two lipogenic transcription factors, sterol regulatory element-binding transcription factor 1c (SREBP1c) and peroxisome proliferator-activated receptor γ (PPARγ), which can promote acne by increasing the proliferation of acroinfundibular keratinocytes and the biosynthesis of lipids in the sebaceous glands [98,99]. In SEB-1 sebocytes, IGF-I promotes sebaceous adipogenesis by increased mRNA expression of SREBP1 through the activation of the PI3K/Akt signaling pathway [100]. Also, activated mTORC1 promotes the expression of survivin, an inhibitor of apoptosis protein family, recently found to be activated in the skin of acne patients, causing acne and acne scars [101]. In contrast, nuclear FoxO1 inhibits mTORC1 in a TSC2-dependent manner by inducing the expression of sestrin3 to activate AMPK, the major negative regulator of mTORC1 (Fig. 5) [102]. Cytoplasmic FoxO1 can bind to the C-terminus of TSC2, thereby dissociating the TSC1/TSC2 complex and inducing the activation of mTORC1 [97]. Currently used anti-acne drugs also show therapeutic efficacy via the nuclear localization of FoxO1 and inhibition of mTORC1 [103,104]. Nuclear FoxO1 and FoxO3a in the sebaceous glands of patients with acne are upregulated by isotretinoin, a drug for acne treatment [105].

Taken together, these observations outline the roles of FoxO1 in the development and severity of acne, and indicate that activating FoxO1 in the sebaceous glands may be an effective approach to improve the acne symptoms.

## 6 Conclusion

Based on the *in vitro* and *in vivo* studies, the importance of FoxOs in maintaining proper skin health is quite clear; however, the activation of FoxOs leads to either beneficial or unfavorable effects depending on the type of skin disorder (Table 1). In skin pigmentation, the activation of FoxO3a and FoxO6 inhibits melanogenesis by integrating the antioxidant signaling and elevating the intracellular anti-oxidant defense system, respectively. In the wound healing processes, FoxO1 induces the gene expression of *VEGF*, stimulating wound healing in normal skin. Meanwhile, the inhibition of FoxO3 is necessary for wound closure in human primary keratinocytes; however, the underlying mechanism associated with this process needs to be further elucidated. In the case of keloid skin, however, FoxO1 inhibition may be beneficial for wound healing as it decreases the inflammatory response and increases the keratinocyte migration, angiogenesis, and collagen degradation. FoxO1 and FoxO3a may be important factors for acne treatment as their activation was observed in the sebaceous glands of acne patients during the application of an anti-acne drug. Although the mechanism underlying the FoxO3a-mediated anti-acne effect is unclear, FoxO1 may exert its effect partly by the inhibition of the mTORC1 signaling pathway. Some FoxOs act as essential tumor suppressors and control several genes related to cell survival, apoptosis, migration, and invasion in melanoma. Thus, their roles in melanoma are well-documented. The activation of FoxO3a downregulates the inhibitors of apoptosis and promotes the apoptosis of melanoma cells. FOXO3a-SIRT6 regulatory axis inhibits glucose metabolism and tumor cell proliferation in melanoma. FoxO1 and FoxO3a-mediated anti-melanoma activities are also derived from the regulation of miRNAs, including miRNA-135a, miRNA-182, and miRNA-194. Therefore, it appears that FoxOs exert anti-melanoma activities via multiple mechanisms, including the transcriptional regulation of several genes and the control of miRNAs. Taken together, the roles of FoxOs in skin health are clearly evident, although the effects of the activation or deactivation of FoxO differ depending on the skin disorders and the progression of the disease state. Further molecular studies are necessary to understand whether FoxOs play specific roles depending on the types of skin cells and methods of treatment, including the exposure to UV, inflammatory factors, and other environmental factors, to stimulate biological aging.

**Table 1:**
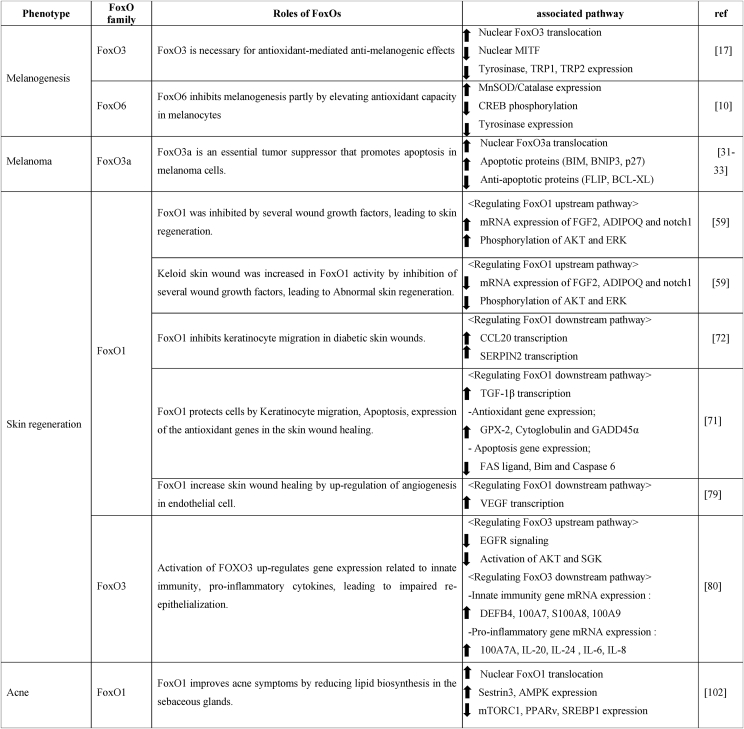
The roles FoxOs in skin health.

## Declaration of competing interest

Please check the following as appropriate:

oAll authors have participated in (a) conception and design, or analysis and interpretation of the data; (b) drafting the article or revising it critically for important intellectual content; and (c) approval of the final version.
oThis manuscript has not been submitted to, nor is under review at, another journal or other publishing venue.
oThe authors have no affiliation with any organization with a direct or indirect financial interest in the subject matter discussed in the manuscript
o The following authors have affiliations with organizations with direct or indirect financial interest in the subject matter discussed in the manuscript:
